# Greater lifestyle engagement is associated with better age-adjusted cognitive abilities

**DOI:** 10.1371/journal.pone.0230077

**Published:** 2020-05-21

**Authors:** G. Sophia Borgeest, Richard N. Henson, Meredith Shafto, David Samu, Cam-CAN, Rogier A. Kievit

**Affiliations:** 1 MRC Cognition and Brain Sciences Unit, University of Cambridge, Cambridge, United Kingdom; 2 Department of Psychology, University of Cambridge, Cambridge, United Kingdom; 3 Cambridge Centre for Ageing and Neuroscience (Cam-CAN), University of Cambridge and MRC Cognition and Brain Sciences Unit, Cambridge, United Kingdom; University of Lleida, SPAIN

## Abstract

Previous evidence suggests that modifiable lifestyle factors, such as engagement in leisure activities, might slow the age-related decline of cognitive functions. Less is known, however, about which aspects of lifestyle might be particularly beneficial to healthy cognitive ageing, and whether they are associated with distinct cognitive domains (e.g. fluid and crystallized abilities) differentially. We investigated these questions in the cross-sectional Cambridge Centre for Ageing and Neuroscience (Cam-CAN) data (N = 708, age 18–88), using data-driven exploratory structural equation modelling, confirmatory factor analyses, and age-residualized measures of cognitive differences across the lifespan. Specifically, we assessed the relative associations of the following five lifestyle factors on age-related differences of fluid and crystallized age-adjusted abilities: education/SES, physical health, mental health, social engagement, and intellectual engagement. We found that higher education, better physical and mental health, more social engagement and a greater degree of intellectual engagement were each individually correlated with better fluid and crystallized cognitive age-adjusted abilities. A joint path model of all lifestyle factors on crystallized and fluid abilities, which allowed a simultaneous assessment of the lifestyle domains, showed that physical health, social and intellectual engagement and education/SES explained unique, complementary variance, but mental health did not make significant contributions above and beyond the other four lifestyle factors and age. The total variance explained for fluid abilities was 14% and 16% for crystallized abilities. Our results are compatible with the hypothesis that intellectually and physically challenging as well as socially engaging activities are associated with better crystallized and fluid performance across the lifespan.

## Introduction

Cognitive abilities are known to decline with age [1,2]. The extent to which leading an active lifestyle can slow down this decline has been debated in the literature, with some studies associating physical, intellectual and social activities with cognitive and neural health while others did not find such relationships [3–5]. Here, we address three open questions regarding the possible associations between lifestyle and cognitive age-adjusted abilities.

First, the relationship between lifestyle and cognition has predominantly been studied by assessing lifestyle activities separately (e.g. by focusing on physical health *or* social engagement, but rarely both). Previous studies which have assessed various aspects of lifestyle have tended to rely on separate linear regressions [6–8], mediation analyses [9] or sum scores [10,11] for their analyses, limiting the extent to which the multidimensionality of people’s lives can be captured, and possible complementary benefits of lifestyle detected. Thus, unless these factors are analysed conceptually and mathematically simultaneously, it remains an open question as to whether individual lifestyle factors will ‘sum up’ to demonstrate incremental benefits, or rather be redundantly associated with better outcomes (see also Kremen et al., 2019 [12]). Our structural equation modelling approach, outlined below, addresses this gap in the literature by offering several benefits compared to previous approaches. First, we model both cognitive and lifestyle factors as latent variables, which abstracts away from individual variables whilst reducing measurement error associated with simple sum scores. Latent variables widen the interpretability of lifestyle-cognition associations to activity *types* (for instance ‘social activity’) instead of *individual* activities (e.g. ‘attending church’). Moreover, we model multiple lifestyle factors within the same large healthy population, allowing us to compare effect sizes. Most uniquely, our structural model captures the *simultaneous* effect of multiple latent lifestyle factors on cognitive lifespan differences, allowing us to investigate whether associations of specific lifestyle domains remain after taking into account distinct, but correlated, lifestyle factors.

Second, little is known about whether different aspects of cognition are associated differently with lifestyle engagement. Following a distinction first made by Cattell [13], cognitive abilities can, at their broadest level, often be grouped into fluid and crystallized abilities (although newer, more detailed conceptualizations are available [14] we focus on fluid and crystallized for their importance in theories of cognitive ageing). Fluid intelligence refers to the ability to solve novel problems in the absence of task-specific knowledge or experience. It predicts important life outcomes such as expected income or work performance [15]. Age produces a marked impairment in fluid intelligence; a decline that begins in early adulthood (see Schaie (1994) for a review [16]). Moreover, recent findings have demonstrated that individual declines in fluid intelligence are highly correlated with individual declines in the ability to live and function independently [17]. Crystallized intelligence, on the other hand, refers to acquired knowledge about the world (such as vocabulary) and shows more modest changes with age than fluid intelligence, typically declining only in old age (i.e. after the late sixties; 2,6,8,9). One open question, addressed here, is whether crystallized and fluid abilities, known to differ in their lifespan trajectories, also benefit differently from measures associated with better cognitive ageing.

Third, it has been difficult to reliably identify those lifestyle activities that enhance cognitive reserve as is demonstrated by the considerable heterogeneity of findings in the literature [18]. This is likely to be due to at least two reasons. One concerns the large diversity of lifestyle variables that have been assessed, with studies differing on the types of activities that make up, say, social engagement. A second explanation is the variable and often imprecise definition of ‘healthy ageing’ in cross-sectional studies. For instance, many cross-sectional studies rely on classifying groups of people according to their absolute performance on cognitive tests (e.g., 27,28). In such an approach, older individuals who score an arbitrary number of standard deviations above a task mean are labelled ‘healthy’, ‘successful’, or in some cases even ‘super’ agers [19–23], while those beneath this cut-off point are considered to age only ‘normally’ or ‘poorly’. Here, we conceptualize ‘healthy aging’ as a matter of ‘age-adjusted cognitive abilities’ by using a simple continuous age-residualized measure, which we describe in more detail below. This measure avoids the drawbacks of arbitrary statistical cut-off points and dichotomisation [24], and allows for a natural conceptualization of age-adjusted cognitive abilities, namely whether an individual is performing better or worse than would be expected at her age.

### The present study

Although enhanced physical, mental and social lifestyle components have all been associated with healthier cognition, these effects have predominantly been investigated in isolation (e.g. by looking at physical health *or* social engagement, but rarely both). A simultaneous analysis of these associations would shed more light on the possible complementary benefits of various aspects of people’s lives. Moreover, understanding if lifestyle is associated differently with crystallised and fluid cognition is important in order to guide effective interventions. We therefore investigated the simultaneous associations between various aspects of lifestyle and both fluid and crystallized age-adjusted abilities. We used a large (N = 708) age-heterogeneous population-based sample from the Cambridge Centre for Ageing and Neuroscience (Cam-CAN), employing age-residualized measures of cognition, data-driven exploratory structural equation modelling and confirmatory factor analysis. Note that as our data are cross-sectional rather than longitudinal or intervention-based, we cannot resolve whether there are direct (causal) associations, nor rule out reverse causation, nor assess whether there are third variables such as genetic influences which induce shared covariance [25]. These limitations are further discussed in the discussion.

Our main hypothesis was that more than one lifestyle factor would, even in joint models, be a significant predictor of cognition. This reflects our assumption that people’s lives are multidimensional, wherein many aspects of lifestyle collectively affect cognition, and that our modelling approach aimed to capture this multidimensionality. A second hypothesis was that cognition would best be captured by a two factor model, to reflect fluid and crystallized abilities. Since this was a descriptive (as opposed to experimental) study, we did not have hypotheses about individual analysis steps (such as the ESEM factor loadings) or the relative strength of individual lifestyle factors in the joint models.

## Methods

### Participants

Participants were drawn from the Stage 2 sample of the Cambridge Centre for Ageing and Neuroscience (Cam-CAN) dataset, described in more detail elsewhere [26,27]. Exclusion criteria included low Mini Mental State Exam (MMSE; 24 or lower; [28]), poor hearing (failing to hear 35dB at 1000 Hz in either ear), poor vision (below 20/50 on Snellen test; [29], poor English knowledge (non-native or non-bilingual English speakers), self-reported substance abuse, and serious health conditions that affect participation (e.g. self-reported major psychiatric conditions, current chemo/radiotherapy, or a history of stroke). 708 people (359 women, 349 men) were recruited, including approximately 100 people in each decile (age range 18–88, M = 53.4, SD = 18.62). Participants provided a wide range of cognitive measures and questionnaire data, summarized below and in Table 1. Ethical approval for the study was obtained from the Cambridgeshire 2 (now East of England-Cambridge Central) Research Ethics Committee. Participants gave full informed consent. The raw data can be acquired by applying for access through the Cam-CAN data portal (https://camcan.mrc-cbu.cam.ac.uk/). All code used in the paper is available via this repository: https://osf.io/7n4d6/

**Table 1:** Description of cognitive behavioural tasks.

Cognitive Domain	Cognitive Task	Task Description	Descriptive Statistics (Mean, SD, Range, Missingness)	References
Executive Function	Fluid Intelligence	Cattell Culture Fair Test, incl. nonverbal puzzles involving series completion, classification, matrices, and conditions.	M = 31.8	
			SD = 6.76	
			Range = 11–44	
			Missing = 6.8%	
	Multitasking (Hotel Task)	Perform tasks in role of hotel manager: write customer bills, sort money, proofread advert, sort playing cards, alphabetise list of names. Total time must be allocated equally between tasks; there is not enough time to complete any one task.	M = 3.07	Shallice & Burgess, 1991
			SD = 1.74	
			Range = 0.2–9.6	
			Missing = 7.1%	
Language Functions	Spot the Word	Involves presenting an individual with pairs of items comprising one word and one non-word, for example, ‘flonty–xylophone’, the individual is required to point to the real word in the pair.	M = 53.58	Baddeley, Emslie & Nimmo-Smith, 1993
			SD = 5.39	
			Range = 24–60	
			Missing = 0.42%	
	Sentence Comprehension	Listen to and judge grammatical acceptability of partial sentences, beginning with an (ambiguous, unambiguous) sentence stem (e.g., “Tom noticed that landing planes…”) followed by a disambiguating continuation word (e.g., “are”) in a different voice. Ambiguity is either semantic or syntactic, with empirically determined dominant and subordinate interpretations	M = 0.89	Rodd, Longe, Randall, & Tyler, 2010
			SD = 0.07	
			Range = 0.46–1	
			Missing = 11.4%	
	Picture-Picture Priming	Name the pictured object presented alone (baseline), then when preceded by a prime object that is phonologically related (one, two initial phonemes), semantically related (low, high relatedness), or unrelated	M = 0.78	Clarke, Taylor, Devereux, Randall, & Tyler, 2013
			SD = 0.09	
			Range = 0.5–0.94	
			Missing = 8.3%	


	Verbal Fluency	Mean of Letter (phonemic) fluency and animal (semantic) fluency task. For phonemic fluency task, participants have 1 min to generate as many words as possible beginning with the letter ‘p’. For semantic fluency task, participants have 1 min to generate as many words as possible in the category ‘animals’.	M = 20.56, SD = 5.34	Lezak, Muriel, & Deutsch, 1995
			Range = 6–37.5	
			Missing = 0.28%	
	Proverb Comprehension	Read and interpret three English proverbs.	M = 4.53	Hodges, 1994
			SD = 1.63	
			Range = 0–6	
			Missing = 7.5%	
Emotional Processing	Face Recognition	Given a target image of a face, identify same individual in an array of 6 face images (with possible changes in head orientation and lighting between target and same face in the test array)	M = 22.88	Benton, 1994
			SD = 2.36	
			Range = 14–27	
			Missing = 7.2%	
	Emotion Expression Recognition	View face and label emotion expressed (happy, sad, anger, fear, disgust, surprise) where faces are morphs along axes between emotional expressions.	M = 8.66	Ekman & Friesen, 1976
			SD = 1.09	
			Range = 3.33–10	
			Missing = 7.1%	

Memory	Visual Short-Term Memory	View (1–4) coloured discs briefly presented on a computer screen, then after a delay, attempt to remember the colour of the disc that was at a cued location.	M = 2.43	Zhang & Luck, 2008
			SD = 0.59	
			Range = 0–3.5	
			Missing = 7.3%	
	Story Recall	Listen to a short story, recall freely immediately after, then again after a delay, and finally answer recognition memory questions. Delayed recall measure used here.	M = 12.88	Wechsler, 1999
			SD = 4.31	
			Range = 0–24	
			Missing = 0.14%	
Motor and Action Function	Choice Motor Speed	Time-pressured movement of a cursor to a target by moving an (occluded) stylus under veridical, perturbed (30°), and reset (veridical again) mappings between visual and real space.	M = 0.19	
			SD = 0.06	
			Range = 0.05–0.85	
			Missing = 7.34%	
	Choice Motor Coefficient of Variation	Standard deviation divided by mean of reaction time of choice motor speed. Reflects the relative measure of variability.	M = 1.84	
			SD = 0.38	
			Range = 0.86–2.98	
			Missing = 7.34%	

### Cognitive variables

13 cognitive tasks were used to assess five broad cognitive domains, which are summarized in Table 1 (for more detail, see [26,27]). The cognitive domains assessed were executive functions, memory, language functions, motor and action function and emotional processing.

### Lifestyle variables

We included a broad set of 23 lifestyle measures from the Cam-CAN dataset, which were collected via a series of different questionnaires, summarized in Table 2. Eight lifestyle variables were obtained during the Home Interview, an extensive face-to-face interview conducted at Stage 1 of Cam-CAN data collection. The remaining variables were obtained during Stage 2. Measures of physical activity, depression and sleep were assessed via the physical activity energy expenditure (PAEE) questionnaire, the Hospital Anxiety and Depression Scale (HADS; [30] and the Pittsburgh Sleep Quality Index (PSQI; [31]), respectively. The remaining 12 lifestyle variables were taken from the Lifetime of Experiences Questionnaire (LEQ; [32]), which measures a broad range of cognitively stimulating experiences and activities during three life phases: youth, 13–29 years; mid-life, 30–64 years; and late-life, 65 years onwards. Within each phase, further details about activities “*specific”* to that time of life (e.g. education in youth) were solicited, as well as *“non-specific”* activities applicable to any phase (e.g. socialising). The LEQ therefore provides information about current life experiences for all participants, as well as retrospective information about previous life experience for participants in their mid- and late-life phases. Usually, this information is reflected in one specific and one non-specific sum score for each stage of life. In this study, however, we focused on a more fine-grained and consistent (across our participants) scoring procedure. First, we define our measure of education as the young-age specific score, derived from the UK’s National Career Service categories and multiplied by number of years at each category. Second, we included only *current* non-specific activities depending on the age of the individual, as we wanted to focus on contemporaneous activities, and allow consistent data across our full age range [33]. Third, as our core goal of this study was to understand which *kind* of lifestyle activity is most strongly associated with age-related cognitive differences, we obtained separate scores for a subset (7) of the non-specific questions, rather than calculating the usual sum-score. As these seven questions (see Table 2) cover a range of lifestyle activities, individual scores for each question gave us more precision in determining their covariance to other lifestyle factors. Non-specific activities were assessed through the same seven questions during youth, mid-life and late-life, addressing participation in i) travel, ii) social outings, iii) playing a musical instrument, iv) artistic pastimes, v) physical activity (mild, moderate, vigorous), vi) reading, vii) learning or speaking a second language. In addition, participants were asked whether their typical day included any of the following four activities: i) internet use, ii) strategic games (e.g. chess, bridge, cards), iii) prayer/religious activity, iv) brain training games. All non-specific scores were scaled to a score from 0–5.

**Table 2:** Description of lifestyle variables. The grouping into ‘lifestyle factors’ is the result of the factor analysis outlined in more detail below.

Lifestyle Factor	Variable	Description/Question	Descriptive Statistics	Reference
**Education/SES**	Income	What is the average total income before tax received by your household? (1–5)	M = 2.83	HI^1^
			SD = 1.49	
			Range = 1–6	
			Missing = 0.14%	
	Smoking habits	category of smoking based on self-report questions (1–3)	M = 1.03	HI
			SD = 0.97	
			Range = 0–3	
			Missing = 1.4%	
	TV watching**	How much TV do you watch per week?	M = 2.2	HI
			SD = 1.47	
			Range = 0–7	
			Missing = 61.9%	
	Body Mass Index (BMI)	Weight (kg) / Height^2^ (m^2^)	M = 25.78	HI
			SD = 4.59	
			Range = 16.75–48.32	
			Missing = 17.2%	
	Travel	Did you travel to any of the following continents between the ages of 13–30 years?	M = 2.3	LEQ^2^
			SD = 1.25	
			Range = 0–5	
			Missing = 12.01%	
		(9 options available)		
	Instrument	How often are you practising or playing a musical instrument?	M = 1.97	LEQ
			SD = 1.22	
			Range = 0–5	
			Missing = 12.01%	
	Language	How often do you practise speaking, reading, writing or learning a second language?	M = 1.89	LEQ
			SD = 1.26	
			Range = 0–5	
			Missing = 12.01%	
	Years of education	Sum score derived from the UK’s National Career Service categories, multiplied by number of years at each category	M = 3	LEQ
			SD = 2.49	
			Range = 0–13.29	
			Missing = 12.3%	

**Physical Health**	Internet	Does your typical day include internet use?	M = 3.39	LEQ
			SD = 1.89	
			Range = 0–5	
			Missing = 12.01%	
	Exercise^+^	Please give the typical number of hours per week you spend in sports and physical activities. Divided into mild, moderate and vigorous activities.	M = 3.43	LEQ
			SD = 1.02	
			Range = 0–5	
			Missing = 12.01%	
	Systolic Blood Pressure	Mean systolic blood pressure of three samples	M = 120.08	HI
			SD = 17	
			Range = 78.5–186	
			Missing = 18.1%	
	Physical activity	Total physical activity energy expenditure (PAEE) calculated from self-report ACTMETS (kJ/day/kg)	M = 4.29	HI
			SD = 2.19	
			Range = 0–17.71	
			Missing = 11.9%	
**Mental Health**	Depression	Hospital Anxiety and Depression Scale (HADS)	M = 2.82	[30]
			SD = 2.58	
			Range = 0–17	
			Missing = 0.56%	
	Quality of sleep	Pittsburgh Sleep Quality Index (PSQI)	M = 5.41	[31]
			SD = 3.68	
			Range = 0–22	
			Missing = 5.4%	
	Alcohol consumption	Amount of alcohol used weekly	M = 3.29	HI
			SD = 1.37	
			Range = 0–5	
			Missing = 3.9%	
	Self-Health	Self-reported health. 4-point scale; 1 = excellent 4 = poor	M = 1.87	HI
			SD = 0.69	
			Range = 1–4	
			Missing = 0.28%	

**Social Engagement**	Exercise^+^	Please give the typical number of hours per week you spend in sports and physical activities. Divided into mild, moderate and vigorous activities.	M = 3.43	LEQ
			SD = 1.02	
			Range = 0–5	
			Missing = 12.01%	
	Social outings	How often might you make an outing to see a family member, friend or group of friends?	M = 3.66	LEQ
			SD = 1.08	
			Range = 0–5	
			Missing = 12.01%	
	Religious Activities	Does your typical day include prayer / religious activities?	M = 2.2	LEQ
			SD = 1.33	
			Range = 0–5	
			Missing = 12.01%	
	Social Mean Score	Derived from 13 question sub-section of Home interview	M = 2.32	HI
			SD = 0.6	
			Range = 0–4.18	
			Missing = 0%	

**Intellectual Engagement**	Reading	Does your typical day include reading?	M = 4.68	LEQ
			SD = 0.92	
			Range = 0–5	
			Missing = 12.01%	
	Brain Training Games	Does your typical day include brain training games (e.g. Computer or Nintendo)?	M = 1.7	LEQ*
			SD = 1.2	
			Range = 0–5	
			Missing = 67.8%	

	Strategic Games	Does your typical day include strategic games (e.g. Chess, Bridge, Cards)?	M = 1.55	LEQ
			SD = 0.98	
			Range = 0–5	
			Missing = 12.01%	
	Artistic Pastime	How often do you practise or develop an artistic pastime (e.g. drawing, painting, sculpture, creative writing, acting, etc.)?	M = 2.09	LEQ
			SD = 1.48	
			Range = 0–5	
			Missing = 12.01%	

1 = Home Interview (Cam-CAN); 2 = Life Experience Questionnaire [32]

* Only older participants were asked this question (N = 228)

** This question was completed in a take-home questionnaire by a subset of the sample (N = 270)

+ The LEQ exercise question cross-loaded onto *Social Engagement* and *Physical Health* in the CFA model and is thus included twice in this table

### Exploratory structural equation model (ESEM)

In order to obtain a data-driven categorization of our cognitive and lifestyle variables, we used a relatively novel technique called exploratory structural equation modelling (ESEM; see [34]). ESEM integrates confirmatory factor analysis (CFA) and structural equation modelling (SEM) to provide confirmatory tests of a priori factor structures. We used the package psych (version 1.7.8; 41) in R-Studio 1.0.153 (R version 3.4.2) [35]. The method is described in more detail in the S1 File.

### Age-residualized cognitive abilities

After computing the best age-related trajectories, we calculated indices of age-adjusted cognitive abilities in each domain. For this, we separately regressed fluid and crystallized factor scores on age, retaining the residual score for each participant and factor. Each residual score thus reflects the difference between the participants’ observed and her age-predicted factor scores. Because the residuals were obtained from a curve reflecting age-related differences, they do not represent the difference between a participant’s score and the *overall mean*, but rather of the mean expected for the participant’s age (thus, the *age-adjusted mean*). Although these scores will still correlate with raw scores within each domain, these residuals adjust for age-expected declines, allowing, for example, an 80-year old person with a relatively low absolute score to be considered *cognitively healthier* than a younger individual with a higher score. Residualized fluid and crystallized cognition therefore serve as our measure of age-adjusted cognitive abilities in further analyses. Similar measures have been proposed to quantify brain structure adjusted for calendar age, [36] and psychosocial functioning adjusted for the severity of adverse childhood experiences [37]. We tested for the assumption of homoscedastic residuals using the Breusch-Pagan test to check if the variability of the residuals increased across the lifespan. Where appropriate we also computed robust regressions to ensure heteroscedasticity did not affect our inferences.

### Confirmatory factor analysis (CFA)

In the second step of our analyses, we used a set of simpler confirmatory factor analyses (CFA models) to a) achieve stable model estimation and b) facilitate detailed model comparisons. CFA is a multivariate statistical procedure that allows the researcher to specify the number of latent and observed constructs in order to test how well the former are captured by the latter. Translating our ESEM solutions to CFA models allowed us to formally test more parsimonious models that remove negligible cross-loadings, and to assess overall model fit using a more conventional range of model fit indices. Although such a two-step procedure is ideally performed on two independent subsamples of the data, this was not feasible given the necessity to balance between model complexity, sample size and stable convergence. To decrease the risk of overfitting, we constrained the range of unnecessary cross-loadings to zero. Although one-step, or factor score regression approaches [38], are generally considered preferable, challenges with convergence and model estimation precluded such approaches here. As such, we specified CFA’s separately for each domain and used estimated factor scores in the second stage. All models were fit using Lavaan 0.6–1.1203 [39]. Prior to model fitting, one variable with very large variance (Multitasking, measured in milliseconds) was rescaled by dividing by 100 to avoid convergence problems. All models were fit using maximum likelihood estimation with robust (Huber-White) standard errors and a scaled chi square test statistic [39]. Missing data, reported in Tables 1 and 2, were accounted for using Full Information Maximum Likelihood method in Lavaan, which allowed us to estimate factor scores for all individuals, including those with partially missing data and yields unbiased estimates under the assumption of missing at random or missing completely at random [40].

Model fit was inspected using the chi-square test of exact fit. Given the considerable sample size which yields high statistical power to reject the test of perfect fit even with modest deviations, we also report the Root Mean Square Error of Approximation (RMSEA) and its confidence interval, the Comparative Fit Index (CFI) and the Standardized Root Mean Square Residual (SRMR). Good fit was defined as approximately RMSEA < 0.05, CFI > 0.97 and SRMR < 0.05, acceptable fit as approximately RMSEA = 0.08–0.05, CFI = 0. 95–0.97, SRMR = 0.05–0.1 [41]. To examine the robustness of the CFA model we refit the subsequent path model in a Bayesian model selection framework [42] using Bayesian regression.

Finally, we examined the degree to which lifestyle factors made *specific* contributions to fluid versus crystallized cognitive differences. To do so, we refit the models while imposing equality constraints on the lifestyle paths. In other words, we compared a model where the effects of lifestyle factors are estimated individually for each of the two cognitive domains, to a more parsimonious model where the path coefficients are assumed to be identical for fluid and crystallized healthy ageing. If the effects of lifestyle factors are equal for both cognitive domains, then one would expect an equality constrained model (where the effects of lifestyle factors on cognitive domains are presumed to be equal) to fit better. However, if certain lifestyle factors have stronger, or weaker, effects on each domain, then one would expect a model that estimates all structural paths freely to fit better.

### Exploratory analyses

We performed a series of exploratory analyses to assess the presence of i) an interaction effect of age and lifestyle using a median split and ii) sex effects.

## Results

### Exploratory structural equation model

**Fig 1:**
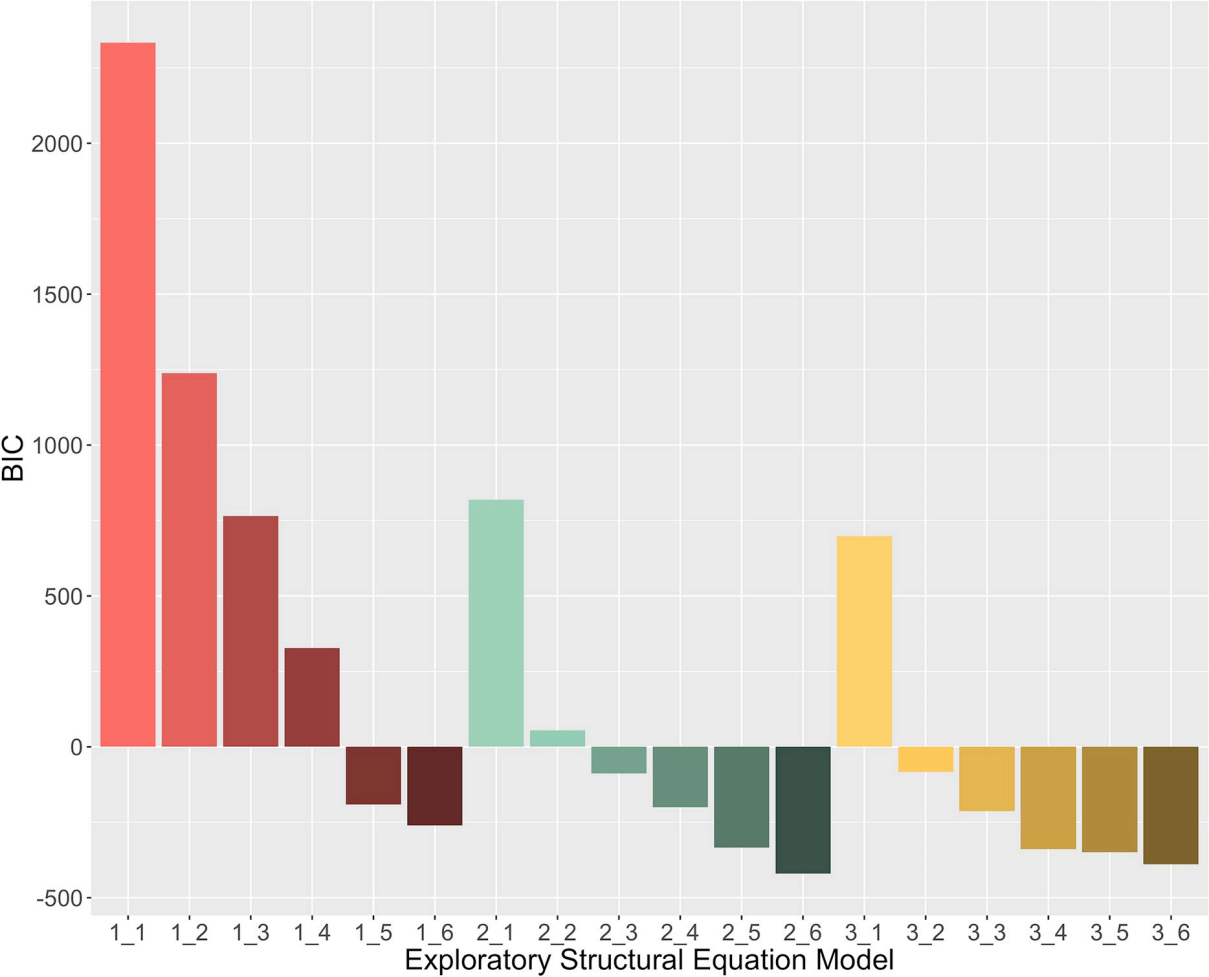
Exploratory structural equation model results. Y-axis reflects Bayesian Information Criterion (BIC) measure of model fit; X-axis labels consist of two digits separated by an underscore (e.g. 2_4), where the first refers to the number of cognitive latent variables, and the second to the number of lifestyle latent variables. Model 2_6 has the best overall fit, then Model 3_6; however, Model 2_5 was selected for further examination due difficulties interpreting the sixth lifestyle factor in the 2_6 and 3_6 models.

The sample-size adjusted BIC scores are shown in Fig 1 (the first number in each model name refers to the number of cognitive variables, and the second number refers to the number of lifestyle variables). The ESEM analyses revealed that, generally, two- and three factor models of cognitive abilities fit the data substantially better than a one factor model. The three factor solutions had marginally better fit than the two factor solutions (e.g., ΔBIC = 13.55 for the 2_5 versus 3_5 model). However, we opted for a two factor solution for theoretical reasons, as the two factor solution closely resembled the canonical distinction between *fluid* and *crystallized* abilities, in line with Cattell [13] and a large body of body of work on cognitive aging [43–45]. Moreover, we note that, in the two factor cognitive model, although the strongest factor loading on the first ‘fluid’ factor is the Cattell test, it includes a relatively large, and broad, number of cognitive abilities, several of which are beyond the traditional remit of pure fluid intelligence [46].

With regards to the lifestyle variables, a purely data driven solution suggested a six-factor lifestyle model (e.g., ΔBIC = 86.54 for the 2_5 vs 2_6 model). However, closer inspection showed that the sixth factor induced a range of hard to interpret cross-loadings, suggesting that it (in both the two- and the three- cognitive factor solution) did not contain information that could be distinguished from the other factors in a meaningful way. For reasons of parsimony and theoretical interpretability, we therefore selected the five-factor solution for further examination.

### CFA: Cognitive model

**Fig 2:**
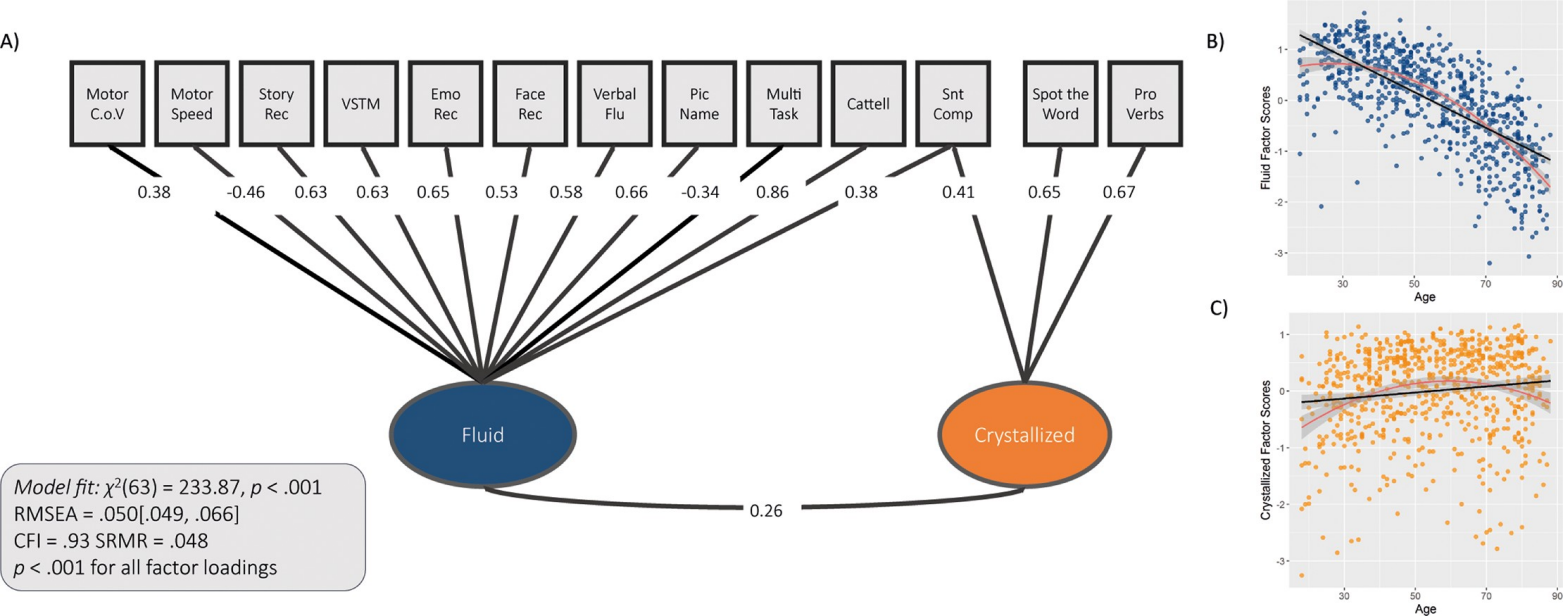
Confirmatory factor model. A) Cognitive CFA. For multitasking and motor speed, lower scores indicate better performance (hence the negative factor loadings). B) Fluid factor scores for each participant. Fluid abilities decline with age. C) Crystallized factor scores for each participant; crystallized abilities show slight increase and then decrease. All parameters shown are fully standardized.

First, we fit the cognitive data with a two-factor model that mirrors the canonical distinction between *crystallized* and *fluid* abilities [13]. One notable exception was that this model required a single data-driven cross-loading for the *sentence comprehension* task, which may reflect the nature of the task as a combination of being knowledge-based (whether a sentence is grammatical) and benefiting from fluid ability. This cognitive measurement model, shown in Fig 2A, fit the data adequately: *χ*^2^ = 233.87 (N = 708), df = 63, p <0.001, RMSEA = 0.057 [0.049 0.066], CFI = 0.93, SRMR = 0.048, suggesting that the cognitive data were well captured by a two-factor model.

Next, we extracted factor scores for all individuals to examine the most appropriate lifespan trajectory for each domain (linear or quadratic). As expected, fluid and crystallized factors showed different lifespan patterns. Scores on the fluid latent variable showed a strong age-related decline, with a modest acceleration of this decline in old age (Fig 2B), consistent with the best-fitting model including a quadratic component (BIC _Quadr_ = 1391.15, BIC _Lin_ = 1458.09, BIC _Cubic_ = 1393.17). Scores on the crystallized latent variable were less variable across the lifespan, with a slight increase until middle age but suggestion of decline in old age (Fig 2C), again consistent with a quadratic component (BIC _Quadr_ = 1676.27, BIC _Lin_ = 1696.91, BIC _Cubic_ = 1678.06).

### Age-residualized cognitive abilities

**Fig 3:**
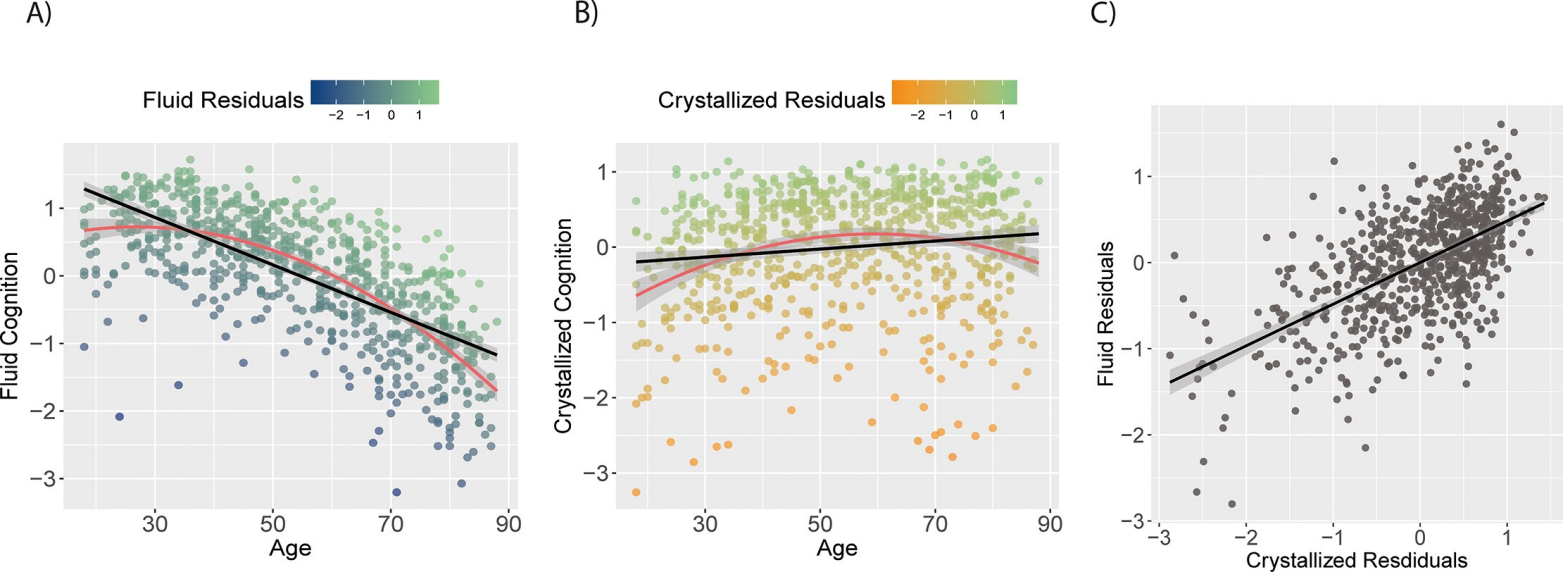
Age adjusted residuals. Residuals as measure of healthy cognitive ageing. A) Crystallized residuals, B) fluid residuals, C) correlation between crystallized and fluid residuals; r(706) = .59, p < .001.

Age-residualized measures of fluid and crystallized abilities (shown in Fig 3) were significantly positively correlated (Pearson’s *r* = 0.59 [0.53 0.63], df = 706, *p* = < 0.001). The median (age 55) split analysis showed that the Gf-Gc correlation of residuals did not differ significantly for the two age groups (*z* = 0.8, *p* = 0.42).

### CFA: Lifestyle model

**Fig 4:**
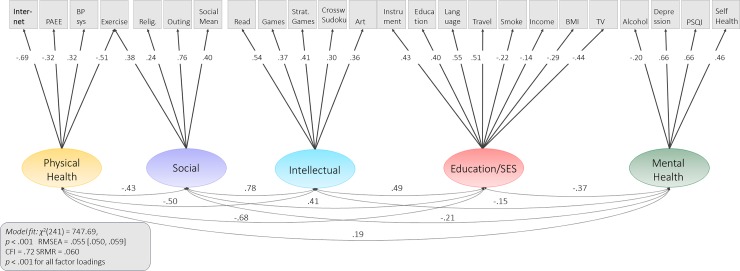
Lifestyle CFA. Following the factor loadings obtained via the ESEM, 24 broad lifestyle variables loaded onto five latent lifestyle variables: mental health, social engagement, intellectual engagement, education/SES and physical health. All parameters shown are fully standardized. All but three lifestyle factor loadings (income, internet usage and alcohol) were in the expected direction.

Next, we examined the lifestyle domains in more detail. To do so, we used the ESEM results to specify a simpler (fewer cross-loadings) CFA that captured the observed variables across five latent factors (Fig 4). Based on the pattern of loadings, we refer to these five latent variables as follows: i) *Education*/*Socio-Economic Status* (SES), ii) *Physical Health*, iii) *Mental Health*, *iv) Social Engagement*, *v) Intellectual Engagement*. Education/SES consisted of eight variables, namely years of education, income, language, travel, smoking, TV watching and instrument playing. Physical Health consisted of systolic blood pressure, internet usage, the PAEE score and the LEQ exercise score. Mental health was captured by alcohol usage, depression, self-reported health and sleep quality. The factor loadings of Intellectual Engagement were reading, brain training games, strategic games, Sudoku/Crossword and the degree of engagement in artistic pastime. Lastly, Social Engagement was characterized by religious activity, social outing, the social activity score from the Home Interview and the LEQ physical exercise score. Note, the labels of the factors are for convenience and based on the strongest loadings–some include factor loadings on variables are not canonically associated with the construct. As was the case for the cognitive CFA, this model therefore required one data-driven cross-loading for the LEQ exercise variable, which may reflect that fact that many physical activities (e.g. basketball, hiking) include significant social aspects. The model showed adequate fit to the data in most respects: *χ*^2^(241) = 747.69 (N = 708), p <0.001, RMSEA = 0.055 [0.050 0.059], CFI = 0.720, SRMR = 0.060, although the CFI is lower than preferable, likely due to the modest factor loadings of some variables. Given the nature of the observed scores (see Table 2), higher scores in Social and Intellectual Engagement and SES/Education reflect *more* engagement and *increased* socioeconomic status, respectively. In contrast, higher scores in the Physical Health and Mental Health factors, however, reflect *poorer* health as their indicators (e.g. blood pressure, mental health symptoms) are considered poor outcomes.

Note that all but three factor loadings were in the expected direction. First, income loaded negatively onto education/SES, where usually higher income is associated with higher SES. One explanation for this could be that Cam-CAN represents a wealthier and more educated sample than the general population, and that in the absence of the “full” range, the effects of income diminish. In addition, although significant, this factor loading of -0.14 was small, and should be interpreted with caution. Second, lower alcohol consumption was associated with poorer mental health, where some might have hypothesized the opposite. However, as was the case for income, the factor loading was small (-0.12), and interpretability is therefore limited. Third, more internet usage was associated with better physical health. We believe that this is largely an SES effect, such that people with higher SES (who, on average, have better physical health) also spend more time browsing the internet.

### Determinants of healthy ageing

#### Separate regressions

**Fig 5:**
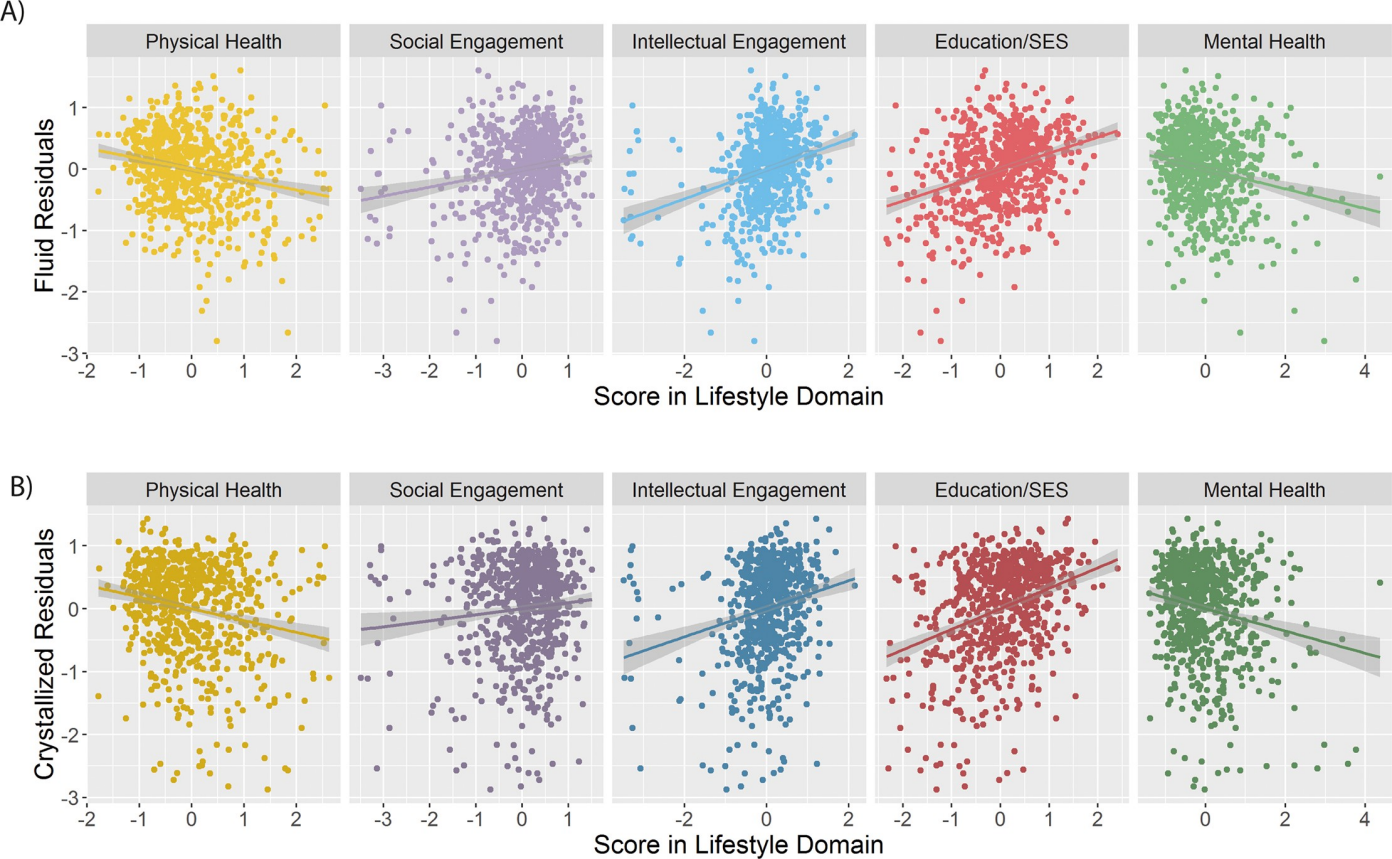
Individual path models. Separate regression results for A) fluid abilities and B) crystallized abilities. All five lifestyle factors were significantly associated with cognitive health across the lifespan.

Next, we investigated the extent to which the five lifestyle factors determined our measures of healthy ageing. As the simultaneous estimation of the measurement models (across cognitive and lifestyle domains) and the structural model (regressing cognitive domains on lifestyle variables) could not achieve robust convergence, we used a two-step procedure. First, we extracted the factor scores for both cognitive factors and computed age-adjusted residuals. Second, we regressed measures of age-residualized fluid and crystallized abilities on the lifestyle factor scores. Doing so, we observed significant associations between each individual lifestyle factor and both fluid and crystallized ageing, as depicted in Fig 5 and Table 3. The strongest associations were those between *Education/SES* and fluid (std β = 0.26) and crystallized cognition (std β = 0.33), followed by *Intellectual Engagement* (fluid std β = 0.24, crystallized std β = 0.22), *Mental Health* (fluid std β = -0.17, crystallized std β = -0.19), *Physical Health* (fluid std β = -0.17, crystallized std β = -0.14) and finally *Social Engagement* (fluid std β = 0.15, crystallized std β = 0.10). All regressions showed modest deviations of the assumption of homoscedastic residuals (all Breusch–Pagan tests χ2>10, df = 1, p<0.01), with a general increase in variability across the lifespan (S1 Fig). To ensure that these heteroscedastic residuals did not affect our inferences concerning lifestyle-cognition associations, we re-estimated all models using a heteroscedasticity-consistent robust sandwich estimator (using the package ‘sandwich’ [47]). As can be seen in Table 3, the parameter estimates and standard errors are virtually identical, suggesting negligible consequences of the heteroscedastic residuals.

**Table 3:** Separate regression results for fluid and crystallized abilities.

Cognitive Domain	Lifestyle Factor	Standardized beta	Standard Error	*p*	R^2^	Robust sandwich *beta*	Robust sandwich SE	*p*
**Fluid Abilities**	Mental Health	-0.16	0.03	<0.001	0.04	-0.16	0.04	<0.001
	Social	0.15	0.03	<0.001	0.03	0.15	0.03	<0.001
	Intellectual	0.24	0.03	<0.001	0.08	0.24	0.04	<0.001
	Education/SES	0.26	0.03	<0.001	0.11	0.26	0.03	<0.001
	Physical Health	-0.17	0.03	<0.001	0.05	-0.17	0.03	<0.001
**Crystallized Abilities**	Mental Health	-0.18	0.04	<0.001	0.04	-0.17	0.04	<0.001
	Social	0.10	0.03	<0.001	0.009	0.79	0.04	<0.001
	Intellectual	0.22	0.04	<0.001	0.05	0.22	0.04	<0.001
	Education/SES	0.33	0.04	<0.001	0.11	0.33	0.04	<0.001
	Physical Health	-0.19	0.04	<0.001	0.04	-0.19	0.04	<0.001

Following recent effect size guidelines [48], we interpret the associations between the lifestyle factors and cognition to range from relatively large (Education/SES) to typical (Intellectual Engagement, Mental Health, Physical Health), with small associations found for Social Engagement. In summary, these findings suggest that having higher levels of education/SES as well as physical and mental health, and partaking in intellectually and socially engaging activities, are all individually associated with better fluid and crystallized cognitive outcomes throughout the lifespan, above and beyond age.

#### Multiple regressions

**Fig 6:**
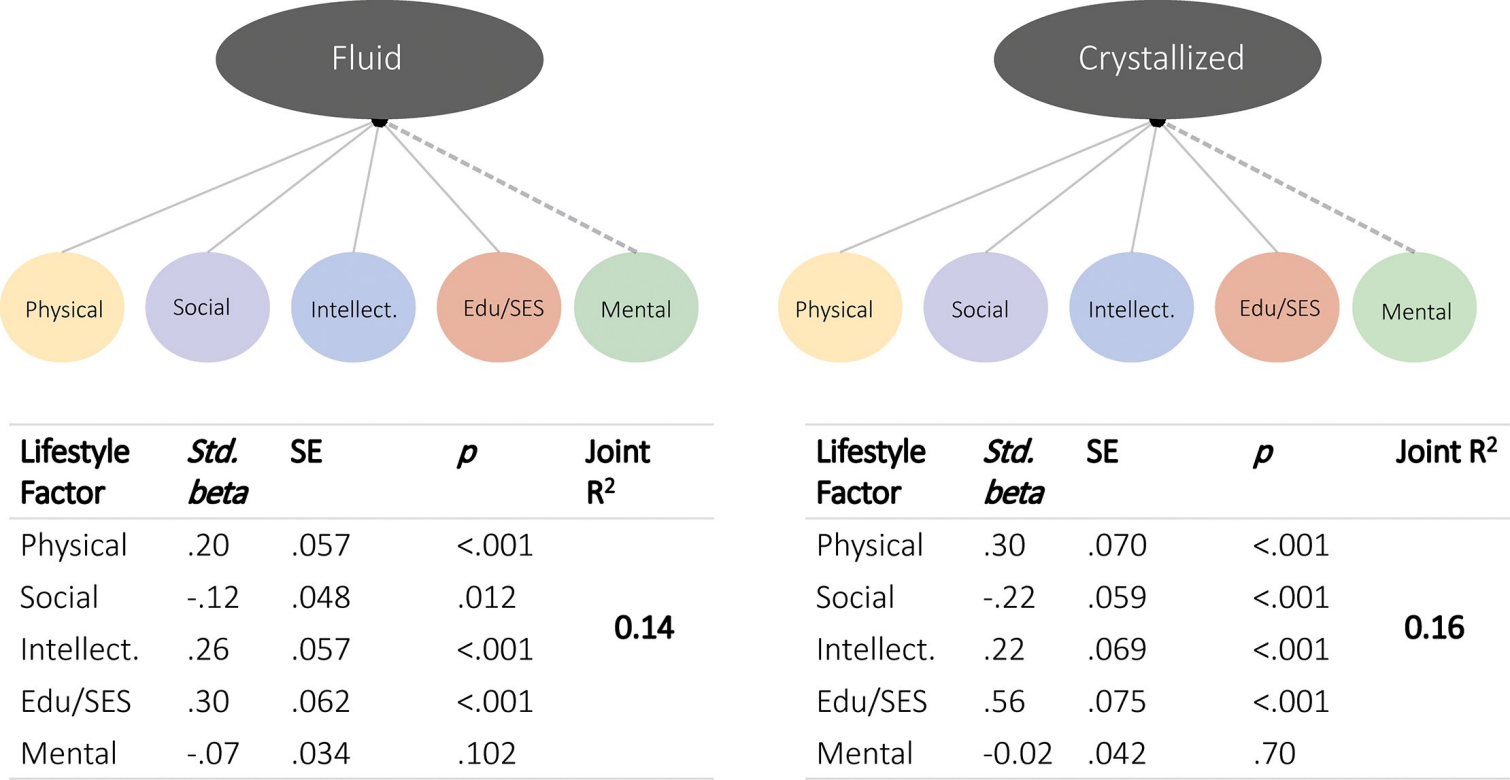
Simultaneous path models. Results of multiple regressions. Four out of five lifestyle factors made unique contributions.

Next, we examined the joint effects of lifestyle factors on healthy cognitive ageing, by simultaneously regressing scores of age-adjusted fluid and crystallized abilities on all five lifestyle factors (Fig 6). Doing so allowed us to examine the degree to which each of the five lifestyle factors make unique contributions to cognitive health. For fluid abilities, Education/SES (std β = 0.30, SE = 0.06, *p* < 0.001), Social Engagement (std β = -0.12, SE = 0.048, p = 0.012), Intellectual Engagement (std β = 0.26, SE = 0.06, p < 0.001) and Physical Health (std β = 0.20, SE = 0.06, p = 0.001), were significant predictors, predicting unique variance in fluid age-residualized abilities, and together explaining 14% of the variance. We found a similar pattern for crystallized abilities, with Education/SES (std β = 0.56, SE = 0.075, p < 0.001), Social Engagement (std β = -0.22, SE = 0.059, p < 0.001), Intellectual Engagement (std β = 0.22, SE = 0.069, p < 0.001) and Physical Health (std β = 0.30, SE = 0.07, p < 0.001) each significant and together explaining 16% of the variance. We did not find evidence that mental health made unique contributions to fluid or crystallized abilities beyond the other lifestyle factors. Notably, in these joint models, the directionality of the effect of Social Engagement changed from positive to negative, while Physical Health changed from negative to positive. These sign inversions may reflect a true conditional association, or rather a quantitative consequence of the dataset and procedure employed here–we discuss these matters in more detail below.

To examine the robustness of this ‘reduced’ model, we refit the model in a Bayesian model selection framework [42] using Bayesian regression. As before in this cohort [49], we used the default, symmetric Cauchy prior with width of $\frac{\surd 2}{2}$ which translates to a 50% confidence that the true effect will lie between −0.707 and 0.707. Doing so yields a Bayes factor for all possible subsets of predictors, thus yielding the model that optimally balances parsimony (excluding unnecessary predictors) with prediction power. In line with the above frequentist model, this comparison (full set of model comparisons shown in S2 Fig) revealed that the best model for both fluid and crystallized abilities included Education/SES, Social Engagement, Intellectual Engagement and Physical Health (but not Mental Health).

Regarding the specificity of the contributing of lifestyle to crystallized versus fluid abilities, we found that the freely estimated model fit marginally better (Δχ2 (5) = 13.92, *p* = .016), suggesting small differences in path estimates. Closer inspection of the parameter estimates showed that this difference was driven almost exclusively by SES, which has a stronger association with crystallized abilities (standardized beta: .56) than with fluid (.38).

#### Exploratory analyses

Our final set of analyses investigated whether there was evidence for an interaction of age or sex with our lifestyle factors: in other words, whether any of the lifestyle factors increase or decrease in importance for cognitive health. First, we performed a multi-group model based on a median age split (median = 55 years), which suggested imposing equality constraints across age group did not adversely affect the associations between lifestyle and cognitive outcomes (Δχ2 (5) = 3.799, *p* = .58). We then tested for the presence of sex effects, which again found that the joint model could be equally constrained across sexes without a notable drop in model fit, Δχ2 (10) = 12.96, p = .23. This suggests that the beneficial associations between lifestyle and cognitive health are similar across age and for both sexes.

## Discussion

### Summary of main findings

In a large lifespan cohort with a broad set of measures, we examined the associations between healthy cognitive ageing and potentially modifiable lifestyle factors. We observed that, in isolation, better physical and mental health, increased social and intellectual engagement and higher levels of education/SES were significantly associated with age-residualized crystallized and fluid cognition (i.e. cognitive abilities higher than those expected for one’s age).

Three out of five lifestyle factors showed typical effect sizes, with Education/SES having a strong, and Social Engagement a small association, respectively [48]. Individual lifestyle domains have previously been correlated with cognitive health in old age and our bivariate results provide further evidence for this relationship. However, as described in the introduction, few studies have investigated combinations of lifestyle factors in a way that allows for statistical inferences regarding their complementary effects (e.g., studies that use five separate linear regressions to investigate the associations between cognition and cognitive and social activity, physical activity, diet, alcohol consumption and smoking [6]). Here, when all lifestyle factors were incorporated into the same model, social and intellectual engagement as well as physical health made independent contributions to fluid and crystallized age-adjusted abilities, above and beyond the effect of education/SES. These relationships were robust across age and sex, and highly similar for fluid and crystallized domains, suggesting general effects, rather than effects specific to cognitive domain. Importantly, social, physical and intellectual activities are potentially modifiable. Assuming they are causally related to cognitive health, interventions to increase them may help boost the cognitive reserve that appears to support independent functioning in old age.

In both the linear regressions and the joint models, the strongest associations were those between education/SES and cognitive health. This ties in well with the literature: for example, a recent systematic review comprising over 130,000 individuals [18] showed that the relationship between education/SES and healthy ageing was reported in 20 of the 25 included studies. One possible explanation is the notion of cognitive reserve, which suggests that education and occupational attainment are driving the brain’s reserve capabilities [50]. Arguably, however, a person’s education or socio-economic status are difficult to alter, particularly later in life. Our finding that physical health and intellectual and social engagement are associated with cognitive health above and beyond education/SES therefore offers further support for the promise that potentially modifiable activities also contribute to cognitive reserve.

One key contribution of this manuscript, echoing recent calls [12], is the *simultaneous* inclusion of multiple lifestyle factors, in order to better understand their relations and independent contributions. Doing so, we show that four of our five lifestyle factors (all except mental health) contribute uniquely in explaining individual differences in cognitive outcomes. Interestingly, two of the path estimates, namely social and physical, changed sign: While they were, as expected, positively associated with outcomes in isolation, the sign of the association changed in the presence of other, collinear predictors. Both substantive and statistical explanations (which are not mutually exclusive) of these patterns are possible, and we outline both below.

Firstly, we found that social activities became negatively associated with cognitive performance. A possible interpretation is that high levels of social activity which are devoid of intellectual activity may be associated with poorer outcomes. For example, social and intellectual activities may tend to co-occur in people (e.g., frequently meeting with family to play games), but once the intellectual component is accounted for, the remaining types of social activity may actually be detrimental to cognitive ability (e.g., drinking alcohol regularly with friends). Further research using more refined lifestyle measures is needed to address this possibility.

Secondly, in the simple regressions we observed that better physical health was associated with better cognitive outcomes–but this association changed in sign in the full model. The simple association is in line with several other papers, including intervention studies, which have suggested that physical activity reliably reduces the risk of cognitive impairment [51–54]. However, not all studies observe the same pattern—the UK Whitehall II study found no evidence between physical activity and subsequent 27 year cognitive decline [55], and Gow et al. [14] found that mid-life intellectual and social activities, but not physical activity, were associated with late-life cognitive health [3]. Notably, sign reversals need not be counterintuitive. For example, in the same Cam-CAN sample, Fuhrmann et al. observed strong associations such that *low* diastolic blood pressure (usually associated with lower overall blood pressure) was associated with worse neural health–but *only* when the model also included systolic blood pressure [56]. This pathway thus captured the conditional effect of a large difference between systolic and diastolic blood pressure, known as ‘pulse pressure’ often associated with (precursors to) diabetes and other medical conditions. Similarly, there may be indirect conditional pathways which substantively explain the sign inversion.

Alternatively, there are more purely quantitative explanations of these sign flips. It is well-known that high collinearity between predictors (here Intellectual Engagement and Social Engagement r = .61; Physical Health and Education/SES r =.-68) inflates the standard errors of the parameter estimates, which can produce changes in sign of the mean [57,58]. However, this increase in standard error would normally render tests on mean no longer significant, which is not the case here (and the standard errors for these paths in the full model were not especially large). More likely is that our findings reflect a type of ‘reversal paradox’ [59]. This phenomenon can occur when parts of a causal chain (i.e. both antecedents and consequences) are incorporated in the same model, inducing–especially in observational data with correlated predictors–reversals of path estimates depending on the nature of the predictors included. In this light, it is worth considering the ‘reverse causation’ hypothesis of Kremen et al. [12]: They state that many of the protective effects of individual differences in lifestyle factors (such as greater cognitive and social engagements, and even education) are themselves the *consequence* of early life differences in cognitive ability.

In the absence of direct access to underlying causal mechanisms generating the data, we cannot conclusively say which of the above explanations are most plausible. As such converging lines of evidence from longitudinal studies, interventions and multivariate approaches will be required to understand the true aetiology of these effects. However, it unambiguously demonstrates the importance of *simultaneous* assessment of multiple lifestyle-cognition associations, if we wish to better understand the complex lifespan process of risk and resilience factors.

The effect of mental health, while significant in univariate analyses, disappeared in the joint models. We interpret this as an important null-finding, suggesting that the association between mental health (measured, in this paper, as an emergent latent construct that was measured by depression, quality of sleep, alcohol consumption and self-reported health) and cognitive health is either less strong compared to other lifestyle factors, or fully explained by co-occurrence with other lifestyle factors. This finding differs from those of other cross-sectional studies, which found associations between depression and poorer cognitive function in old age [60–62]. However, this discrepancy can, in part, be explained by the high degrees of comorbidity between depression and dementia, given that the above studies (unlike the current one) included participants with mild cognitive impairment (MCI) and/or Alzheimer’s disease (AD). Indeed, a longitudinal study that employed latent growth models showed that, when participants with MCI and AD were removed from the models, the association between cognitive health and depression disappeared [63].

We observed no significant difference of the lifestyle-cognition associations for crystallized compared to fluid age-adjusted abilities; both were captured best by models including education/SES, social engagement and intellectual engagement. We interpret this to suggest that lifestyle is likely to benefit cognition in a global, rather than specific manner. This might have important ramifications for the interpretation of cognitive intervention studies, which often fail to find positive transfer effects. Assessing cognition on latent and global levels, rather than by performance on individual tasks might be, as has been suggested elsewhere [64], a more desirable statistical approach.

### Strengths and limitations

A strength of our analyses is the inclusion of an unusually broad and rich set of lifestyle and cognitive variables in a large lifespan cohort. Uniquely, this allows us to directly compare the relative strength of associations of distinct lifestyle factors within the same healthy population.

The most important limitation of this study is that the data investigated here are cross-sectional. For this reason, although our findings align well with other work, we cannot make direct causal inferences regarding the observed associations, as they may be explained by a variety of causal pathways, included omitted third causes. Moreover, as noted above, causality may flow in both directions–better cognitive health may facilitate the desire, as well as capacity, to maintain an active life in old age [8]. These issues can be addressed to some extent by longitudinal studies, and most directly by interventional studies. However, it may be all but impossible to engage in a true randomized intervention study of factors as integral to individuals as education, social and intellectual engagement. As such, large observational studies relying on powerful multivariate methodology may offer an imperfect, but nonetheless valuable insight into which lifestyle factors are most likely to have beneficial protective effects in ageing, and therefore provide candidate factors which might be more amenable to intervention studies (as well as advising what other factors should be controlled for in such studies). Moreover, we only examined relationships between current activities and current cognitive abilities: it is possible that many years are needed before lifestyle changes affect cognitive abilities. For example, one’s current lifestyle activities in old-age may be of little value if similar beneficial activities were not conducted earlier in life, consistent with our previous findings using retrospective questionnaires, where people’s activity scores in their current, old age did not make a unique contribution above the same activity scores reported from their previous, mid-life period [33]. Further work is needed to more precisely reveal the temporal development of the beneficial effects of lifestyle engagement on cognitive abilities.

Methodologically, our approach comes with strengths and limitations. The use of exploratory structural equation modelling (ESEM) allowed us to categorize the observed variables in a mainly data-driven fashion–an approach that has the potential to decrease researchers’ subjectivity and selection bias and improve statistical power. However, some loadings of the data-driven lifestyle factors may strike some as counterintuitive. Relatedly, by grouping lifestyle variables into factors, we decrease the specificity of associations of individual variables, and render the hypothetical translation to intervention targets (i.e. to encourage the increase of purportedly beneficial activities) less straightforward. This reflects a general issue, namely that the assessment of lifestyle-cognition associations warrants a trade-off between generalizability and reduction of measurement error (using latent variables) versus specificity and ease of interpretation (using observed variables). The latter approach has led researchers to conclude, for instance, that knitting, doing odd jobs and gardening all reduce the risk of dementia [65]. However, a defence of latent lifestyle factors would posit that such activities are better seen as reflecting a *class* of activities with similar purported beneficial effects. If there is causal efficacy to, say, knitting, then a coherent causal account would likely posit that activities with similar features (subjective enjoyment, social engagement) would lead to similar beneficial accounts. This line of reasoning is implicitly present in intervention studies that focus on e.g. ‘physical activity’, ‘cardiovascular training’ or ‘coordination training’ (rather than ‘walking’ or ‘using a fitness ball’; e.g., [66]). Additionally, even with individual variables, the notion of modifiability of lifestyle factors is not entirely straightforward, since the behaviours and personality characteristics that are amenable to intervention or modification, and the circumstances that enable alterations, have yet to be established. Factors like personality, mood, people’s perception of their abilities, as well as more external limitations including mobility and financial security, are all likely to affect the extent to which people alter the various aspects of their lives. Theory- or prediction-based approaches, such as mixture models or decision-tree based methods [67], might provide useful tools to explore these open questions.

Next, although several indicators of model fit are in the acceptable or good range, the CFI is lower than ideal. As the CFI is an index of comparative fit compared to the null model, a lower CFI often occurs for larger measurement models with moderate to low factor loadings. Although several of our factor loadings are strong (e.g. social outings on the social factor) others are lower (e.g. alcohol consumption on mental health). This is likely a consequence of reporting the best fitting exploratory model, which, in a large lifespan observational sample such as Cam-CAN, is likely to group together variables with only moderately strong relations to each other. In contrast, much more well-established measurement models, refined over multiple cohorts, tend to lead to the selection of only indicators with (very) high loadings. As our goal here is explicitly a descriptive, exploratory factor analysis to reduce a rich sample of indicators to a tractable number of lifestyle factors, such a strategy would not be appropriate, both for reasons of generalizability (modifying the factor structure purely for reasons of fit) and principle (we wish to convey the full richness of the data *including* factor loadings and relationships that perhaps don’t fit pre-existing groupings). More importantly, the regressions (both the simultaneous and individual) show moderate to strong effects, suggesting that despite a subset of relatively weak loadings, the factor scores are separable and predictive of external outcomes. As such, we prefer the model as is, with several model fit indices that are good but with a less than optimal CFI, rather than us modifying the model to simply achieve a better fit. This reasoning is also in line with our objective to use a data-, as opposed to researcher-driven categorization of variable: While an advantage of modifying the measurement model might be (slightly) better model fit, we believe that the advantages of the data-driven approach (i.e. increased objectivity and greater ease of replicability with other datasets and variables) outweigh these concerns.

Finally, because Cam-CAN represents a sample of healthy adults, the generalizability of our findings to other populations remains to be investigated by future research.

## Conclusion

In conclusion, our findings suggest that lifestyle variables can be grouped into distinct but correlated factors. Moreover, these factors vary in the strength of their associations with cognitive health, and make specific, complementary contributions in explaining individual age-related differences. Specifically, we found that education/SES, physical health and social and intellectual engagement, are each simultaneously associated with higher age-adjusted cognitive abilities across the adult lifespan, and these associations are similar in magnitude and direction for two broad cognitive domains (fluid and crystallized). Mental health, although associated when tested with better cognitive outcomes in isolation, did not make unique contributions above the other three lifestyle factors. Because many of the activities included in our models are, in principle, modifiable, our findings have encouraging implications for individuals and public health initiatives alike.

## Supporting information

S1 Fig Test for homoscedasticity. Figure shows modest deviations of homoscedasticity across the lifespan.
(TIF)

S2 Fig Bayesian model selection. Figure shows Bayesian model selection converging with frequentist inferences, with model evidence displayed in descending order.
(TIF)

S1 File (DOCX)
