# Supplementary figures and images for: Greater lifestyle engagement is associated with better age-adjusted cognitive abilities
Source: PLoS One. 2020 May 21;15(5):e0230077. doi: 10.1371/journal.pone.0230077 (PMC7241829; doi:10.1371/journal.pone.0230077)

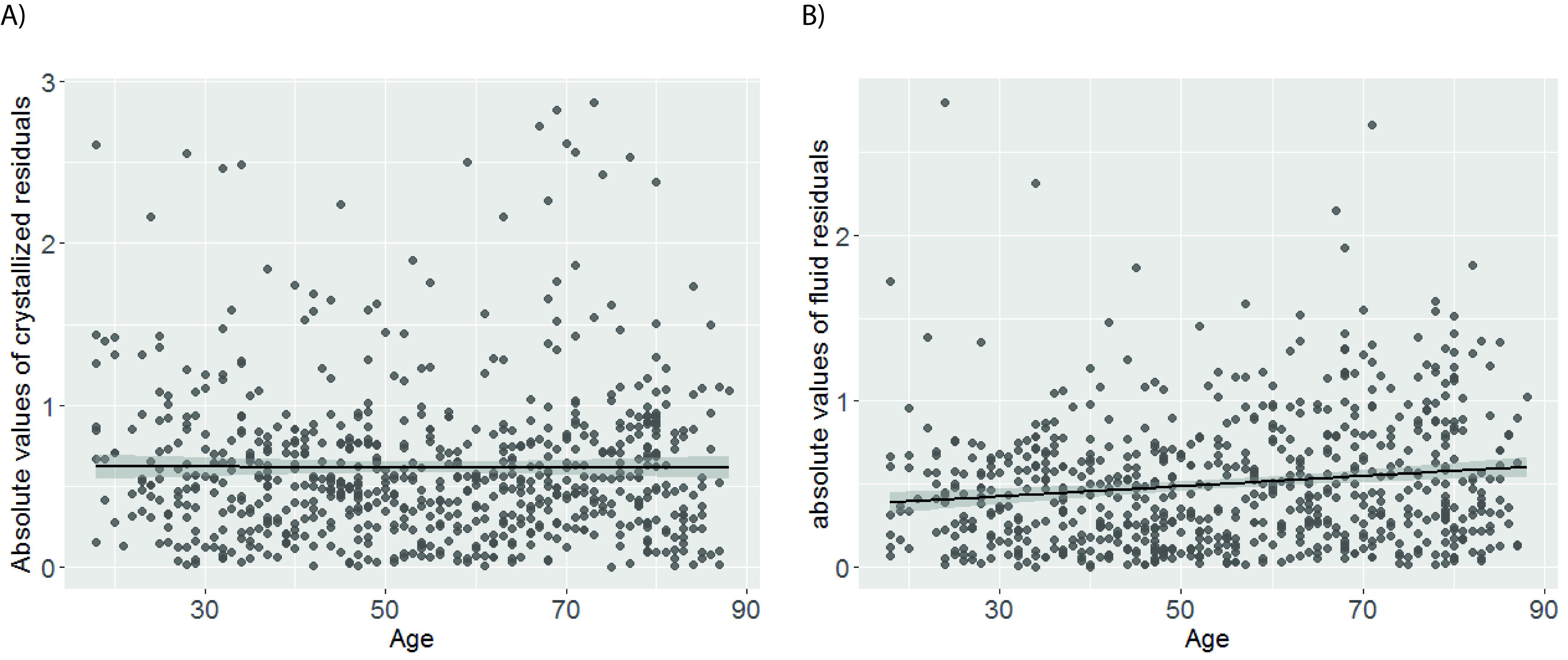

Supplement: S1 Fig — Figure shows modest deviations of homoscedasticity across the lifespan. (TIF) [file pone.0230077.s001.tif]

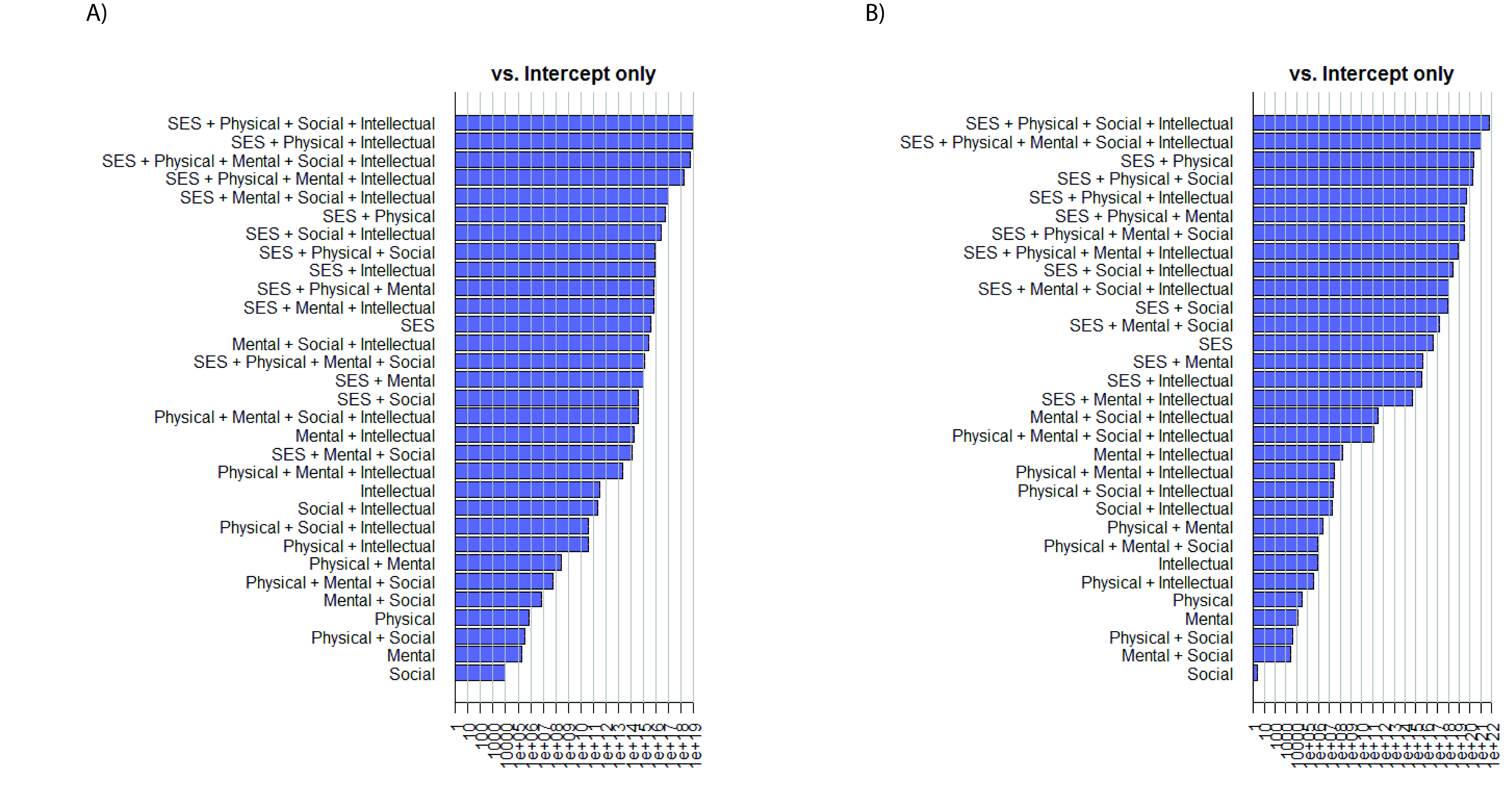

Supplement: S2 Fig — Figure shows Bayesian model selection converging with frequentist inferences, with model evidence displayed in descending order. (TIF) [file pone.0230077.s002.tif]
