# Supplementary material for: Greater lifestyle engagement is associated with better age-adjusted cognitive abilities
Source: PLoS One. 2020 May 21;15(5):e0230077. doi: 10.1371/journal.pone.0230077 (PMC7241829; doi:10.1371/journal.pone.0230077)
Supplement: S1 File — (DOCX) [file pone.0230077.s003.docx]

## **Exploratory Structural Equation Modelling (ESEM) -- further explanation and rationale**

ESEM can be distinguished from CFA mainly in that the former does not require the researcher to define an a-priori factor structure. Based on theory, CFA measurement models specify a number of factor loadings fixed at zero to reflect a hypothesis that only certain factors influence certain factor indicators. The CFA approach of fixing many or all cross-loadings at zero may force a researcher to specify a more parsimonious model than is suitable for the data. Because of this, models often do not fit the data well and there is a tendency to rely on extensive model modification to find a well-fitting model. A commonly used alternative to CFA is Exploratory Factor Analysis (EFA), which solves some of CFA’s challenges in situations of limited measurement knowledge of the researcher and / or a more complex measurement structure. However, EFA cannot be performed while allowing correlated residuals, and it assumes that all measured variables are related to every latent variable. For these reasons, researchers often opt for an ad-hoc procedure that mimics the EFA factor definitions in a SEM model with a CFA measurement specification. This EFA-to-CFA conversion has been shown to be challenging, and can lead to mis-specified models. The main advantage of the ESEM model over other modelling practices is that ESEM incorporates seamlessly the EFA and SEM models. ESEM integrates EFA into SEM (which otherwise relies on CFA measurement models) by estimating the measurement and structural model parts simultaneously. The key difference between ESEM and Principal Component Analysis (PCA) is that the latter is a linear combination of variables, whereas the former (as it relies on factor analyses) is a latent variable measurement model.
